# Supplementary material for: Modeling glioblastoma heterogeneity as a dynamic network of cell states
Source: Mol Syst Biol. 2021 Sep 16;17(9):e10105. doi: 10.15252/msb.202010105 (PMC8444284; doi:10.15252/msb.202010105)
Supplement: Supplementary file 6 — Source Data for Figure 5 [file MSB-17-e10105-s004.zip › Figure5A_sourcedata/GSEA_3071/hallmarks_stateI.GseaPreranked.1623742531165/HALLMARK_ALLOGRAFT_REJECTION.html]

Details for gene set HALLMARK\_ALLOGRAFT\_REJECTION[GSEA]

|  || Dataset | state13071 |
| Phenotype | NoPhenotypeAvailable |
| Upregulated in class | na\_pos |
| GeneSet | HALLMARK\_ALLOGRAFT\_REJECTION |
| Enrichment Score (ES) | 0.3853883 |
| Normalized Enrichment Score (NES) | 1.8533586 |
| Nominal p-value | 0.0026737968 |
| FDR q-value | 0.009293384 |
| FWER p-Value | 0.07 |
Table: GSEA Results Summary

  

Fig 1: Enrichment plot: HALLMARK\_ALLOGRAFT\_REJECTION      
 Profile of the Running ES Score & Positions of GeneSet Members on the Rank Ordered List

  

| PROBE | GENE SYMBOL | GENE\_TITLE | RANK IN GENE LIST | RANK METRIC SCORE | RUNNING ES | CORE ENRICHMENT || 1 | TIMP1 |  |  | 47 | 1.381 | 0.0497 | Yes |
| 2 | THY1 |  |  | 94 | 1.114 | 0.0874 | Yes |
| 3 | F2R |  |  | 105 | 1.067 | 0.1334 | Yes |
| 4 | INHBA |  |  | 140 | 0.941 | 0.1667 | Yes |
| 5 | TGFB1 |  |  | 165 | 0.884 | 0.2003 | Yes |
| 6 | EGFR |  |  | 175 | 0.864 | 0.2372 | Yes |
| 7 | B2M |  |  | 241 | 0.744 | 0.2525 | Yes |
| 8 | IL18 |  |  | 272 | 0.710 | 0.2764 | Yes |
| 9 | TAPBP |  |  | 287 | 0.692 | 0.3041 | Yes |
| 10 | SOCS1 |  |  | 292 | 0.686 | 0.3343 | Yes |
| 11 | TLR3 |  |  | 359 | 0.625 | 0.3439 | Yes |
| 12 | FLNA |  |  | 373 | 0.614 | 0.3683 | Yes |
| 13 | IFNGR1 |  |  | 407 | 0.580 | 0.3854 | Yes |
| 14 | CTSS |  |  | 509 | 0.502 | 0.3793 | No |
| 15 | IL15 |  |  | 635 | 0.439 | 0.3634 | No |
| 16 | GBP2 |  |  | 674 | 0.422 | 0.3718 | No |
| 17 | JAK2 |  |  | 886 | 0.353 | 0.3272 | No |
| 18 | ACVR2A |  |  | 934 | 0.343 | 0.3293 | No |
| 19 | CDKN2A |  |  | 983 | 0.331 | 0.3307 | No |
| 20 | BCAT1 |  |  | 1103 | 0.305 | 0.3104 | No |
| 21 | STAT1 |  |  | 1136 | 0.301 | 0.3149 | No |
| 22 | TAP2 |  |  | 1171 | 0.295 | 0.3186 | No |
| 23 | DEGS1 |  |  | 1178 | 0.293 | 0.3303 | No |
| 24 | ABI1 |  |  | 1200 | 0.288 | 0.3374 | No |
| 25 | DYRK3 |  |  | 1202 | 0.287 | 0.3503 | No |
| 26 | CAPG |  |  | 1222 | 0.285 | 0.3579 | No |
| 27 | GZMB |  |  | 1273 | 0.274 | 0.3560 | No |
| 28 | PSMB10 |  |  | 1375 | 0.256 | 0.3386 | No |
| 29 | CCND3 |  |  | 1482 | -0.255 | 0.3198 | No |
| 30 | EIF5A |  |  | 1905 | -0.306 | 0.2122 | No |
| 31 | ETS1 |  |  | 2086 | -0.332 | 0.1756 | No |
| 32 | MRPL3 |  |  | 2189 | -0.350 | 0.1623 | No |
| 33 | UBE2N |  |  | 2458 | -0.403 | 0.1036 | No |
| 34 | AKT1 |  |  | 2617 | -0.444 | 0.0784 | No |
| 35 | NME1 |  |  | 2669 | -0.463 | 0.0849 | No |
| 36 | HDAC9 |  |  | 2927 | -0.560 | 0.0365 | No |
| 37 | BRCA1 |  |  | 3185 | -0.765 | -0.0024 | No |
| 38 | TGFB2 |  |  | 3251 | -0.852 | 0.0178 | No |
| 39 | IL11 |  |  | 3403 | -1.239 | 0.0311 | No |
Table: GSEA details [plain text format]

  

Fig 2: HALLMARK\_ALLOGRAFT\_REJECTION: Random ES distribution      
 Gene set null distribution of ES for **HALLMARK\_ALLOGRAFT\_REJECTION**
